# An In Vivo Study Using T-scan III Occlusal Analysis System: Does the Extraction Pattern Affect the Final Occlusion in Orthodontics?

**DOI:** 10.7759/cureus.47965

**Published:** 2023-10-30

**Authors:** Rim Fathalla, Hanady Samih, Ahmed Abdel Fattah Ramadan

**Affiliations:** 1 Department of Orthodontics, Faculty of Dentistry, Suez Canal University, Ismailia, EGY

**Keywords:** orthodontic treatment, extraction, bite force, t-scan, occlusion

## Abstract

Background: The assessment of an orthodontic patient's occlusion throughout the treatment and after debonding permits the orthodontist to improve functional occlusion through interventional tooth movements, thus rendering the overall treatment more efficient in terms of stability and masticatory efficiency. This study aimed to evaluate the effect of four first premolar extractions during orthodontic treatment on the distribution of bite force using the T-scan III system (Tekscan Inc., Boston, MA).

Objective: We aim to evaluate the effect of four first premolar extractions during orthodontic treatment on bite force distribution.

Methods: Ten patients (mean age: 16 ± 2.72 years), who would be treated orthodontically with four first premolar extractions to treat their teeth crowding, were selected for this study. The T-scan III system was utilized to measure the occlusal bite force of the patients before and after treatment, and the findings were compared.

Results: There was a non-statistically significant decrease in the occlusal bite force's mean in the arch's anterior segment from 24.45% (± 8.50%) to 14.25% (± 12.93%) after the orthodontic treatment. A non-statistically significant increase in the occlusal bite force in the posterior right segment of the arch from 37.64% (± 18.13%) to 41.65% (± 11.52%) was found after the treatment. The occlusal bite force in the posterior left segment of the arch increased insignificantly from 30.53% (± 20.00%) to 43.95% (± 13.22%). There was an even distribution of bite force on both sides of the arch by the end of the treatment.

Conclusions: Orthodontic treatment helps to achieve a functional occlusal balance by assisting in the uniform distribution of biting force on both sides of the arch. There was no statistically significant change in the distribution of bite force recordings collected before and after orthodontic treatment, indicating that the removal of the four first premolar teeth does not impact the functional aspect of occlusion. The T-scan III system serves as an essential guide during orthodontic treatment to monitor occlusal changes.

## Introduction

Improving masticatory and postural function is one of the primary goals of orthodontic treatment. This, in turn, makes it possible to achieve functional comfort. Because treating complex malocclusions with fixed appliances requires the orthodontist to adjust dental contacts to achieve a new position of equilibrium, an evaluation of the quality of the final occlusion of the cases that have been treated must be performed. This evaluation must consider how well the patient can chew and how stable the teeth are [1].

The occlusal changes in post-orthodontic patients are associated with more temporomandibular disorder (TMD) signs/symptoms than in non-orthodontic patients, as shown in some studies. This was attributed to the prolonged disocclusion time following orthodontic treatment. It was found that orthodontic treatment created fewer working interferences. As the group function occlusion was reported to be more prevalent in the post-orthodontic patients, combined with non-working contacts, this increased the prevalence of TMD [2].

Considering this comes the importance of the functional occlusal relationships that follow tooth movement either during or after orthodontic treatment. The assessment of the patient's occlusion throughout the treatment and after debonding permits the orthodontist to improve functional occlusion through interventional tooth movements when necessary, thus rendering the overall treatment to be more efficient and decreasing the patient's discomfort and optimizing occlusal settling [3].

Nowadays, occlusal forces and occlusal contact data can be precisely evaluated both quantitatively and qualitatively using very thin sensors inserted in the patient's mouth and adapted to the dental arch. These sensors are made of conductive ink, and with loading while the patient bites on them, the electrical resistance of these sensors lessens. These changes in the resistive ink are measured by the system hardware electronics as a change in the digital output voltage. The sensors are connected to a handle, known as the sensor support, whose electronics scan all the digital output voltages, and the system software will analyze the data and display it as a graphical display on the computer's monitor. Therefore, the occlusal bite force and distribution may be readily assessed throughout the arches in real time, dynamically, and in various clinical scenarios by employing computerized instruments such as the T-scan system (Tekscan Inc., Boston, MA) [4].

Therefore, this study aimed to assess how the distribution of bite force would change if premolars were removed during orthodontic therapy.

## Materials and methods

Study sample

Ethical approval (registration number: 2018/105) was obtained from the Research Ethical Committee (REC) of the Faculty of Dentistry, Suez Canal University. The number of samples needed for the study was determined with the help of G* power version 3.9.1.6. [5]. It was established that a sample size of at least 10 patients was required to detect an effect size of 0.88 with a power of 0.8 at a significance level of 0.05. This was the conclusion reached by the researchers. Ten patients seeking orthodontic treatment at the Department of Orthodontics, Faculty of Dentistry, Suez Canal University, were selected for this study. They fulfilled the following criteria to be eligible for participation in the study: age group from 14 to 25 years, a full complement of permanent teeth (except third molars), no previous orthodontic/orthopedic/orthognathic surgical treatment, no history of trauma, no obvious/gross facial asymmetry, and the case analysis indicated the need for extraction of four first premolars to relieve the malocclusion problem. The selected patients had severe crowding more than 7 mm in the arch and had Angle Class I malocclusion. They needed extraction of their four first premolars in order to relieve the crowding and achieve Class I canine and molar relationships. The objectives and methodology were explained to all participants, and written informed consent was obtained.

T-scan occlusion recording before starting the orthodontic treatment

**Figure 1:**
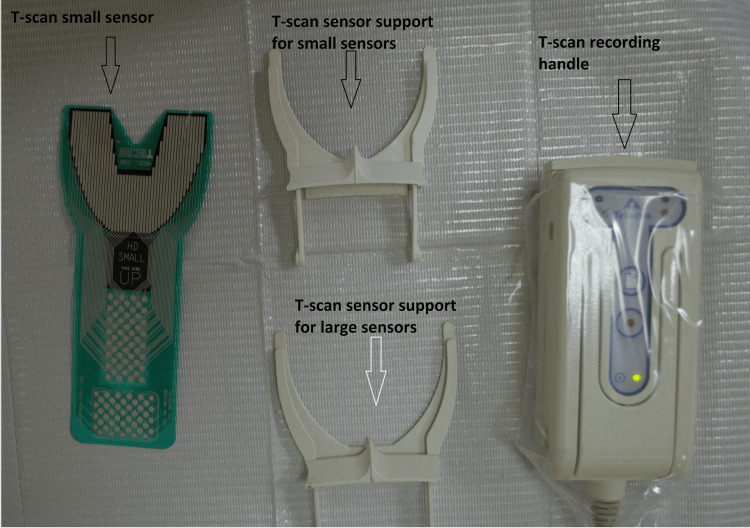
Components of T-scan III: small sensor, sensor support (large and small), and the recording handle

**Figure 2:**
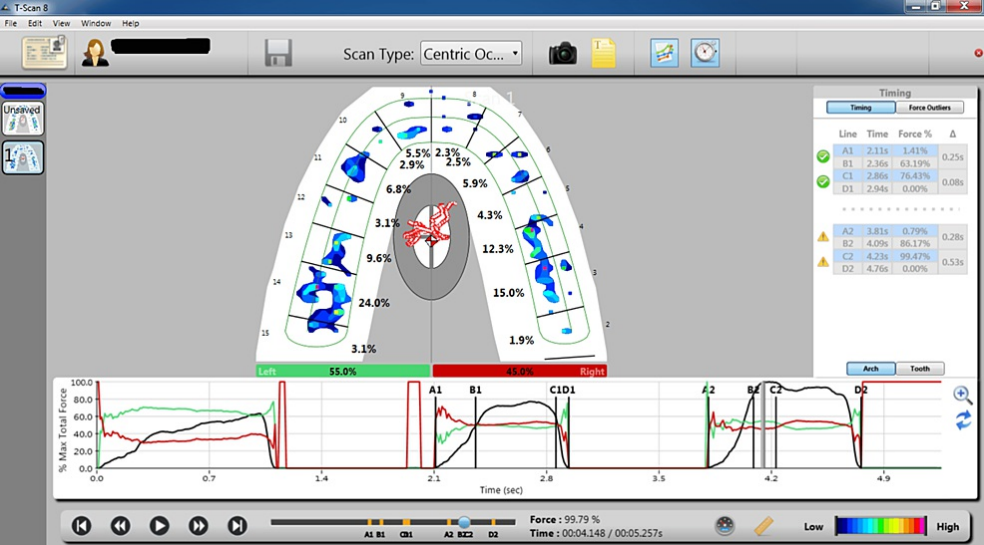
Graphic representation obtained from a T-scan of a patient undergoing orthodontic treatment displaying the 2D, 3D, and force versus time graphs in the quadrant during centric occlusion The timing table was taken before treatment. It displays the distribution of biting force inside and on both sides of the arch.

The T-scan III (Tekscan Inc., Boston, MA) was used to measure the occlusal bite force (Figure 1). The patient was sitting in an upright position without any kind of head support, and the Frankfurt horizontal plane was primarily parallel to the ground. Every patient was instructed to bite down on the sensor, positioned between the arches and in the exact middle of the space between the incisors. When the patient chews on the sensor, the data is processed and shown visually on the computer screen in two or three dimensions [6]. All the patients were repeatedly trained to bite on the sensor in centric occlusion. For every patient, the occlusal bite force was recorded during centric occlusion. The subjects had to clench their teeth three times on the sensor while checking for proper device placement and mandibular centering. The data on the force is shown as a percentage concerning the total loading, divided for each recording into two distinct sectors: right and left (Figure 2).

T-scan occlusion analysis

All of the patients were analyzed with the T-Scan system to obtain their values for the following parameters (Figure 2): force distribution in the patient's arch at maximum intercuspation, levels of occlusal force exerted on the mouth's right and left sides at maximal intercuspation, and the disparity in the percentage of force distributed on the right and left sides of the arch at its highest intercuspation.

The study was done using the mean occlusal bite force derived from the three collected recordings of bite force. The patients were given orthodontic treatment that consisted of extracting their four first premolars and closing extraction space with fixed appliances to achieve optimal occlusion with the therapeutic objectives satisfied. Using the same methodology described earlier, another set of T-scan data was obtained for each participant immediately after removing the fixed appliance at the end of the orthodontic treatment.

Statistical analysis

The data obtained before and after therapy were collated, and then, the appropriate statistical test was utilized to analyze. The Shapiro-Wilk test, bar charts, and descriptive statistics were used to investigate whether the quantitative data were normally distributed. The quantitative variables did not follow a normal distribution and were mostly provided with their mean and standard deviation (SD). Each piece of information was laid up as a percentage. A Wilcoxon signed-rank test was carried out to make a comparison between the records that were obtained before and during orthodontic treatment. The level of significance was determined to be a P value of 0.05. The Statistical Package for the Social Sciences (SPSS) for Windows version 23 (IBM SPSS Statistics, Armonk, NY) was used to perform the analysis.

## Results

Ten patients were enrolled in this study. The distribution between genders was homogenous as shown in Table 1. The Wilcoxon signed-rank test was performed to compare the records taken before and after orthodontic treatment. The significance level was set at a P value of 0.05. All tests were two-tailed. Data were analyzed using SPSS for Windows version 23.

**Table 1:** Baseline characteristics of the sample SD: standard deviation

		Patients (N = 10)
Age (mean (SD))		16.00 (2.72)
Gender (number (%))	Males	5 (50%)
	Females	5 (50%)

There was a non-statistically significant decrease in the occlusal bite force's mean in the arch's anterior segment from 24.45% (± 8.50%)to 14.25%(± 12.93%) after the orthodontic treatment, as shown in Table 2. On the other hand, following the completion of the orthodontic treatment, there was an increase in the occlusal bite force in the posterior right segment that was not statistically significant. This rise was from 37.64% (± 18.13%) to 41.65% (± 11.52%). The occlusal biting force in the left posterior part of the arch increased non-significantly from 30.53% (± 20.00%) to 43.95% (± 13.22%).

**Table 2:** Comparison of the distribution of biting force in centric occlusion between the anterior, posterior right, and posterior left portions before and after orthodontic treatment SD: standard deviation

		Patients' group (N = 10)
Anterior	Before (mean (SD))	24.54 (8.50)
	After (mean (SD))	14.24 (12.93)
	P value	0.093
Posterior right	Before (mean (SD))	37.64 (18.13)
	After (mean (SD))	41.65 (11.52)
	P value	0.327
Posterior left	Before (mean (SD))	30.53 (20.00)
	After (mean (SD))	43.95 (13.22)
	P value	0.575

Table 3 describes the relative (percentage) distribution of occlusal forces on the left and right sides during centric occlusion recorded before and after the orthodontic treatment. Before treatment, the bite force was concentrated on the right side (53.81%) more than the left side (46.19%). After treatment, the bite force was evenly distributed on both sides. No statistically significant difference was found as shown in Table 3.

**Table 3:** Comparison of the relative distribution of occlusal forces on the right and left side of the dental arch during centric occlusion and the difference in percentage force distribution between the right and left sides taken pre- and post-orthodontic treatment SD: standard deviation

		Patients' group (N = 10)
Right	Before (mean (SD))	53.81 (19.55)
	After (mean (SD))	49.19 (11.64)
	P value	0.575
Left	Before (mean (SD))	46.19 (19.55)
	After (mean (SD))	50.81 (11.64)
	P value	0.575
Right-left	Before (mean (SD))	7.62 (39.10)
	After (mean (SD))	-1.62 (23.29)
	P value	0.575

## Discussion

The functional and occlusal alterations resulting from the removal of premolar teeth during orthodontic therapy have not been exhaustively addressed in the published research. Evaluation of the morphological occlusal relationship of the teeth clinically after treatment or through study models is inadequate since a case with clinically satisfied occlusion could be functionally imbalanced while having clinically satisfied occlusion [7]. That is why it is essential to evaluate the occlusion statically and functionally [8].

Occlusal bite force assessment methods and T-scan III

The T-scan III system was used in the current study to assess the distribution of the occlusal bite force. This was done because previous studies [9,10] have shown the importance of T-scan III as a dynamic occlusal indicator in orthodontics.

Distribution of occlusal bite force on teeth

In this study, we found that more forces were concentrated in the posterior regions than the anterior regions, either pre-treatment or post-treatment. This is due to the larger occlusal table of molars and wider occlusal contacts. This finding agreed with the studies of Agbaje et al. [11] and Alkan et al. [12]. Our study showed a decrease in the mean of the occlusal bite force in the anterior segment of the arch. This result is in accordance with the study of Yoon et al. [13], where they demonstrated a decrease in the occlusal bite force after the orthodontic treatment in the non-extraction and extraction groups. In addition, once the orthodontic treatment had been completed, there was an insignificant rise in the occlusal bite force on the posterior right and left sides of the mouth.

Relative distribution of occlusal forces on both sides of the dental arch during centric occlusion

Our study showed that the right side had more force than the left side before treatment, which agreed with Ma et al. [14]. This may be attributed to the fact that the patients had the right side as their preferred chewing side, which is related to the lateral asymmetry of the bite force reported in our study.

A non-significant decrease in the distribution of occlusal bite force on the right side and an increase on the left side occurred after the orthodontic treatment, thus eventually leading to an even distribution of bite force on both sides. This latter finding agreed with Seth et al. [8] and Qadeer et al. [6].

Difference in percentage force distribution between the right and left sides of the arch at maximum intercuspation

The current research demonstrated a great difference between the right and left percentage forces observed at maximal intercuspation before the start of the orthodontic treatment. However, there was a non-significant decrease in the mean difference in percentage force distribution between the right and left sides of the arch after the orthodontic treatment, indicating a more even distribution of the occlusal bite force by the time the orthodontic treatment was complete. As a result, this contributes to the achievement of a more balanced functional occlusion. This is found to agree with Qadeer et al. [6].

Premolar extraction and functional occlusion

The current study found no statistically significant differences between the distribution of occlusal bite force in the patients before and after they underwent extraction of their four first premolar teeth as a part of their orthodontic treatment. This demonstrates that there is no correlation between the extraction of the premolars and the occlusal function. This was confirmed by Choi et al. [15], who concluded in their study that premolar extraction did not induce any decline in the functional aspect of occlusion. This result disagreed with Yoon et al. [13]. The latter research found a tendency for the occlusal function to recover after two years to the pre-treatment level in a group with two premolars extracted. However, in the four-premolar extraction group, it was not fully recovered after two years of follow-up, indicating a decrease in occlusal function. This disagreement might be due to the difference in the sample size and the technique used to register the occlusal bite force. In our study, the T-scan III system was used, and one of its drawbacks is that it does not give an absolute value for the maximum bite force like the Dental Prescale System used in the study by Yoon et al. [13]. However, the T-scan records the relative distribution of bite force in percentages.

Study limitations

Although the current study is the first to assess the distribution of occlusal bite force in orthodontic patients undergoing four first premolar extractions using the T-scan occlusal analysis system, it has some limitations. The small number of patients was the main limitation of this study. Further studies are indeed needed to investigate the occlusal force parameters following different extraction patterns such as upper premolar extraction, lower incisor extraction, and molar extraction. Longer follow-up studies would be beneficial to monitor the occlusal changes during the retention phase.

## Conclusions

From this study, it was concluded that before and after orthodontic treatment, occlusal biting forces are more focused on the posterior teeth than on the front teeth. The orthodontic treatment aids in the even distribution of bite force on both sides of the arch by the end of the treatment in both patient groups, thus achieving functional occlusal balance. The removal of premolar teeth does not have an impact on the functional aspect of occlusion in patients who have had their four first premolars extracted, as there was no statistically significant difference in the distribution of bite force records that were taken prior to and after orthodontic treatment for the patients in this study. The T-scan III system is a key diagnostic tool used to monitor the occlusal changes during the whole orthodontic treatment.
